# Effect of loading history on material properties of human heel pad: an in-vivo pilot investigation during gait

**DOI:** 10.1186/s12891-022-05197-w

**Published:** 2022-03-15

**Authors:** Zhao-lin Teng, Xiong-gang Yang, Xiang Geng, Yan-jie Gu, Ran Huang, Wen-ming Chen, Chen Wang, Li Chen, Chao Zhang, Maimaitirexiati Helili, Jia-zhang Huang, Xu Wang, Xin Ma

**Affiliations:** 1 Department of Orthopedic Surgery, Huashan Hospital, Fudan University, No.12 Wulumuqi Middle Road, Shanghai, 200040 China; 2 Academy for Engineering & Technology, Fudan University, No.220 Handan Road, Shanghai, 200438 China

**Keywords:** Viscoelastic properties, Contact-pressure plate, Dual fluoroscopic system, Continuous loading

## Abstract

**Background:**

This study was aimed to develop a novel dynamic measurement technique for testing the material properties and investigating the effect of continuous compression load on the structural and mechanical properties of human heel pad during actual gait.

**Methods:**

The dual fluoroscopic imaging system (DFIS) and dynamic foot-ground contact pressure-test plate were used for measuring the material properties, including primary thickness, peak strain, peak stress, elastic modulus, viscous modulus and energy dissipation rate (EDR), both at time zero and following continuous loading. Ten healthy pilot subjects, aged from 23 to 72 (average: 46.5 ± 17.6), were enrolled. A “three-step gait cycle” is performed for all subjects, with the second step striking at a marked position on the force plate with the heel to maintain the location of the tested foot to be in the view of fluoroscopes. The subjects were measured at both relaxed (time-zero group) and fatigue (continuous-loading group) statuses, and the left and right heels were measured using the identical procedures.

**Results:**

The peak strain, peak stress, elastic modulus, and EDR are similar before and after continuous load, while the viscous modulus was significantly decreased (median: 43.9 vs. 20.37 kPa•s; *p* < 0.001) as well as primary thicknesses (median: 15.99 vs. 15.72 mm; *p* < 0.001). Age is demonstrated to be moderately correlated with the primary thicknesses both at time zero (*R =* -0.507) and following continuous load (*R =* -0.607). The peak stress was significantly correlated with the elastic modulus before (*R =* 0.741) and after continuous load (*R =* 0.802). The peak strain was correlated with the elastic modulus before (*R =* -0.765) and after continuous load (*R =* -0.801). The correlations between the viscous modulus and peak stress/ peak strain are similar to above(*R =* 0.643, 0.577, − 0.586 and − 0.717 respectively). The viscous modulus is positively correlated with the elastic modulus before (*R =* 0.821) and after continuous load (*R =* 0.784).

**Conclusions:**

By using dynamic fluoroscopy combined with the plantar pressure plate, the in vivo viscoelastic properties and other data of the heel pad in the actual gait can be obtained. Age was negatively correlated with the primary thickness of heel pad and peak strain, and was positively correlated with viscous modulus. Repetitive loading could decrease the primary thickness of heel pad and viscous modulus.

## Introduction

The heel is the first point of contact between the foot and ground during human locomotion, acting as the primary shock absorber to minimize the impact stress transferred to the skeletal system [1–3]. The heel fat pad is histologically composed of honeycombed fat globules formed by clustered fat cells in whorls of fibroelastic septa [4]. The intact configurations of both the adipocyte cluster and fibrous envelop are necessary for the proper functional properties of heel pad [2, 4, 5]. However, many degeneration phenomena, such as aging, diabetes, plantar heel pain, rheumatoid arthritis, deformity, dysvascula of foot, and trauma, may cause significant alterations on the histomorphology of foot pad, involving incrassation of septa, disorganization of septa caused by breaking of collagen bundles and fragmentation of elastin strands, and relative shrinking of adipocytes [1, 2, 6–8]. These alterations, subsequently, would result in further modifications on the biomechanical properties, causing increased stiffness (namely elasticity) of septa, decreased damping ability, and increased vulnerability of tissue to injury [1, 4, 6–9]. To accurately identifying the mechanical parameters of heel fat pad of individual patient, therefore, is essential for possible early diagnosis and treatment for the pathological conditions [10].

Many previous studies have attempted to quantify the viscoelastic material properties either in vitro or in vivo [5, 11–18]. The in-vitro material properties of the tissue cut from plantar pad have been determined with stress-relaxation and compression experiments under uniaxial compressive loading [5, 11, 12]. In-vitro mechanical testing, nevertheless, has been proven to give apparently discrepant properties of heel fat pad compared to in-vivo testing, demonstrating a six-time higher stiffness and a three-time lower energy dissipation rate for in vitro testing [13–15]. This discrepancy has been regarded as a paradox, and the authors concluded that the presence of entire lower leg in in vivo tests indeed influences the measurements [15]. More recently, indentation test have been widely conducted for identifying the viscous properties of heel pad [10, 16]. Using this method, however, the authors primarily aimed to measure the viscous properties of the plantar fat pad for living subjects based on stress-relaxation test. In some later studies, ultrasound elastography approach has also been used to test mechanical properties of plantar soft tissue [17, 18]. However, both of the indentation test and ultrasound elastography approach were not able to replicate the actual loading conditions experienced by the heel when contacting with the ground during dynamic gait cycle. To overcome this limitation, other authors have developed some innovative methods to dynamically measure the material properties of heel pad [19–24]. Ugbolue et al. [19, 20] established a novel deformation and biomechanical assessment method for heel pad of healthy adults based on 3D digital image correlation technology and plantar force plate. The authors simulated the heel raising during gait cycle, and analyzed the influence of gender and dominant foot on the surface deformation and biomechanical properties at the heel pad. De Clercq et al. [21] and Gefen et al. [22] incorporated the fluoroscopy (cine-radiography) and simultaneous pressure test plate beneath the foot to measure the in vivo mechanical properties during dynamic gait. However, using the fluoroscopy, the authours could exclusively capture two-dimension images on the location of heel pad, which would lead to bias on the measured deformation due to the varing shooting angle [21, 22]. The dual fluoroscopic imaging system (DFIS), which could capture two perpendicularly intersected images for reconstructing a three-dimension structure, has been recently used in many situations to help investigate the structural and locational indexes among orthopedic surgeons.

Additionally, it was evident that the loading history has influence on material properties of soft tissues [25–27]. However, few studies focused on the effect of loading history on material properties of heel pad. Thus, the current research was aimed to employ a DFIS investigation integrated with a contact-pressure plate to identify the effect of continuous compression load on the structural and mechanical properties of human heel pad during actual gait.

## Methods

### Inclusion of subjects

In accordance with the Declaration of Helsinki, and upon attaining the ethical approval from the Institutional Review Board of Huashan Hospital, Fudan University, 10 healthy adult subjects (7 male and 3 female), aged from 23 to 72 (average: 46.5 ± 17.6), were enrolled. All subjects received written informed consent at the time before participation. The mean body mass index (BMI) of these participants was 24.6 ± 3.5 (range: 19.2 ~ 31.9) kg/m^2^. Participants with definitively diagnosed pathological conditions that could affect the properties of heel pad, including diabetes, plantar heel pain, rheumatoid arthritis, foot deformity, dysvascula of foot, history of foot surgery, and trauma of foot, would be excluded. Each subject received CT scan on foot before the experiment, for building models of calcaneus that would be used in 2D-3D registration with Mimics Medical 21.0 (Materialize, Belgium).

### Apparatus

**Fig. 1:**
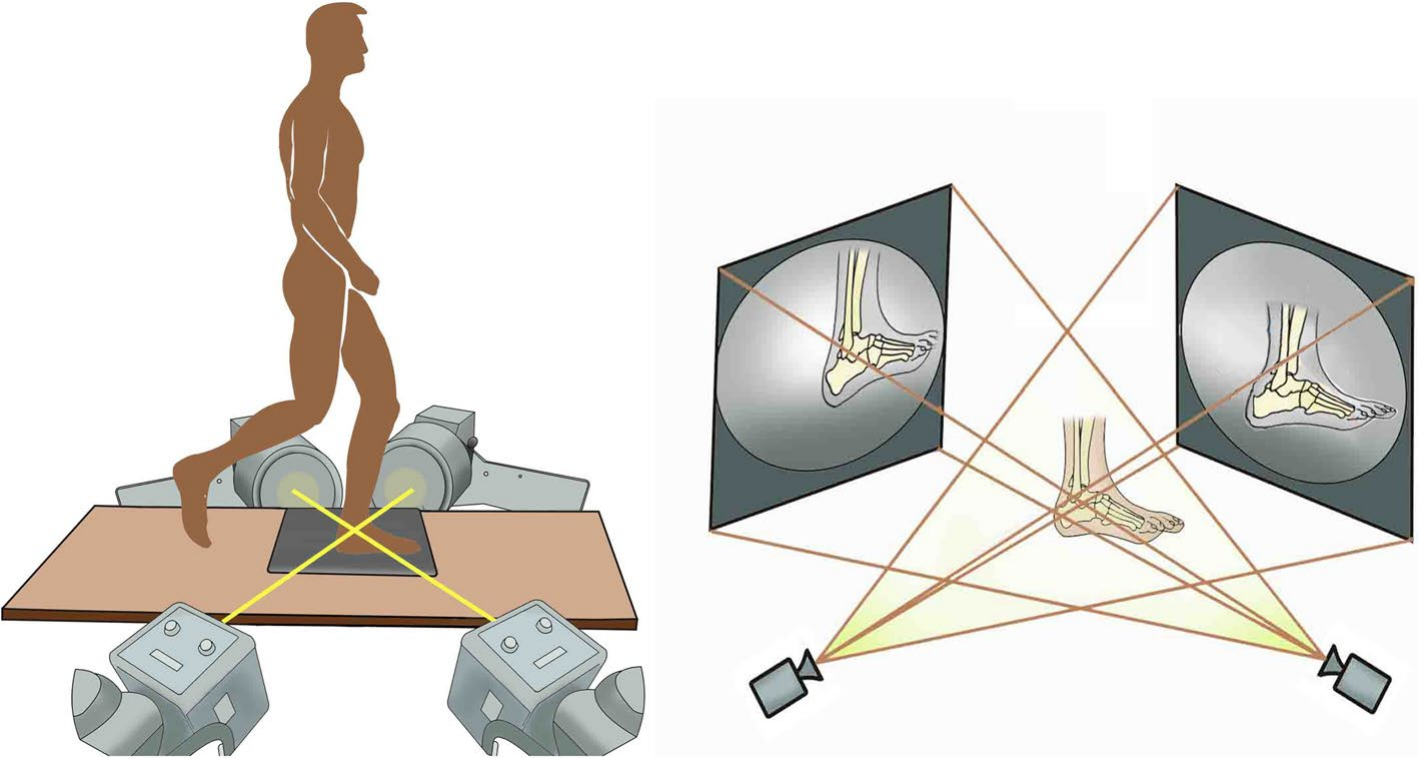
Diagram of the equipment consisted of two orthogonally placed fluoroscopes and dynamic foot-ground contact force plate embedded in the custom gait platform

A diagram of the equipment is presented in Fig. 1. Two C-arm fluoroscopes (BV Pulsera, Phillips Medical, USA) placed orthogonally were utilized for capturing the in vivo compressive strain of the heel pad, with pre-set frame rate of 50 Hz, resolution of 1024 × 1024 pixels and beam energy setting of 75 kV•40 mA. At the same time, a dynamic foot-ground contact pressure-test plate (zebris PDM-XS, 570*400*15 mm, Germany) was embedded in the custom gait platform (with length of 3.5 m and width of 0.8 m) to continuously record the evolution of compressive pressure.

### Experiment design

At beginning of the experiment, a cube calibrator and a pair of retiform calibrators matching the biplane were placed at the intersection of the two fluorescent projections, and placed on the two receivers of the fluoroscopes respectively to obtain calibration images, which would be used for calibrating the distortions of fluoroscopies before model-image registration. Three steel balls were placed on the force plate and fluoroscopic images of balls were collected to mark the spatial position of plate. Following gathering of the calibration and marking images with the aid of software XMAlab (bitbucket.org/xromm/xmalab/), the subjects were allowed to try to walk barefooted on the gait platform with the aid and protection of researchers, until they could locomote deftly and stably without any aid. To eliminate the impact of strain rate on the mechanical properties of heel fat pad, the subjects were required to move with a gait velocity of about 1.0 m/s. A “three-step gait” cycle was performed for all subjects, with the second step striking at a marked position on the force plate with the heel to maintain the location of the tested foot to be in the view of fluoroscopes.

After the participants were familiar with the experiment procedures, they were required to keep foots relax and free of load for one hour. Then the left and right heels were measured using the identical procedures and data were divided to time-zero group. Next, the participants were delivered to continuous loading on the heel pad by sustaining standing or wandering position for 15 min before testing. Afterwards, subjects were taken back to the gait platform to measure the properties of heel pad at fatigue status. The data was divided to continuous-loading group.

### Data processing

The data obtained by DFIS were handled with software Rhinoceros 5.0 (Robert McNeel & Associates, Washington, USA), to get the time-dependent strain of heel pad. The validation test of the 3D kinematic measurement of the calcaneus using 2D-3D registration has been carried out in our previous study [28]. After 2D-3D registration, a plane was defined by centers of three steel balls. The plane was parallel to plate and distance was the radius of the balls. After the 2D-3D registration of each frame, the plane and calcaneus were converted in the form of points to MATLAB (MathWorks, Natick, USA). With the aid of MATLAB, the thickness of the heel pad was equal to the minimum distance between the plane and the calcaneus plus radius of balls. And the thickness measured on the frame, in which skin of heel initially contacts with force plate, was regarded as the primary thickness of heel pad. Strain (*Ɛ*_c_) was change in thickness divided by primary thickness. The corresponding data recorded by contact force plate were exported as xml file. According to the pressure distribution in mid-stance, heel area was selected. Stress (б_c_) was defined as heel-ground contact force of heel area divided by acreage of heel area in each frame. Stress-strain curve, depicting a cycle of loading and unloading process of heel pad, was then generated using strain values and stress values matched according to time points, as well as Strain-time curve. Afterwards, the peak stress and peak strain were calculated at the point with maximal stress and strain in stress-strain curve for each subject. Strain rate (έ_c_) is the tangent at each time point of the Strain-time curve. The corresponding data are fitted to the kelvin-vioght model: б_c_ = −E_c_ - *ηƐ*_c_έ_c_, and the elastic modulus(*E*) and viscous modulus(*η*) were obtained according to the least squares method. The kelvin-vioght model used for calculation are same with that of Gefen et al. [22]. Additionally, the energy dissipation was also calculated from the stress-strain curve, which was defined as the area in the hysteresis loop bounded by the loading and unloading curves. The energy absorption rate (EDR) was finally numerically calculated as the ratio of energy dissipation and the area under the loading curve.

### Statistical analysis

Continuous variables were presented as median as well as the range. Two correlation matrices were generated for properties measured at time zero and following continuous loading. Pearson’s correlation analysis was used to detect the correlation between continuous variables in matrices, and correlation efficient (R) was calculated to depict the magnitude of the correlativity. Additionally, paired box plot was generated to depict the changes of the properties of heel pad after continuous compressive load using paired Wilcoxon test. All of the above statistical analyses were conducted with R version 3.5.0 (Foundation for Statistical Computing, Vienna, Austria).

## Results

**Fig. 2:**
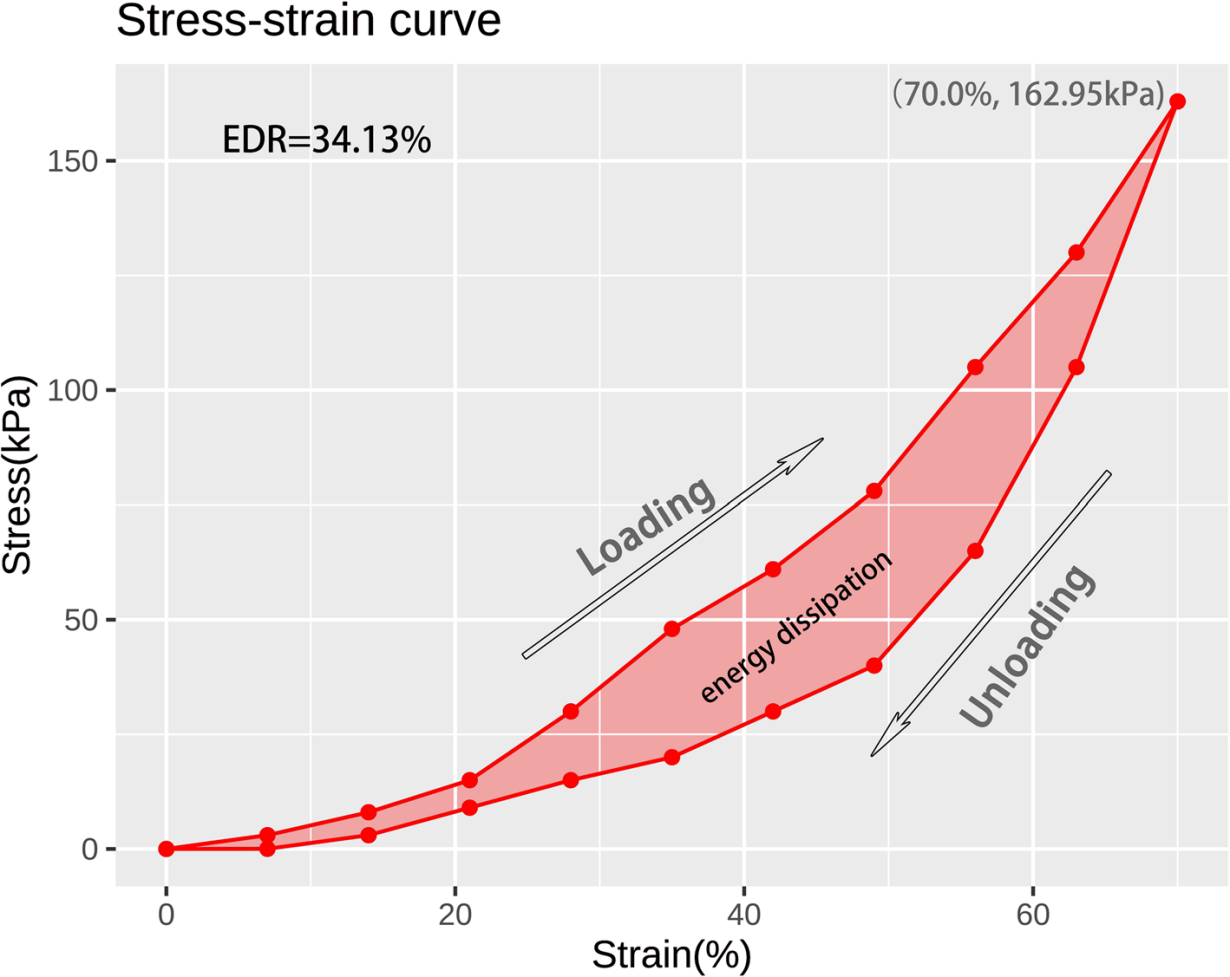
A representative stress-strain curve depicting a cycle of loading and unloading. The energy dissipation was defined as the area between the loading and unloading curves, and the energy dissipation rate was defined as the ratio between energy dissipation and area under loading curve. The point with maximal stress and strain in stress-strain curve represents the peak stress and peak strain. In this individual subject, the peak strain, peak stress and energy dissipation rate were 70.0%, 162.95 kPa and 34.13%, retrospectively

**Fig. 3:**
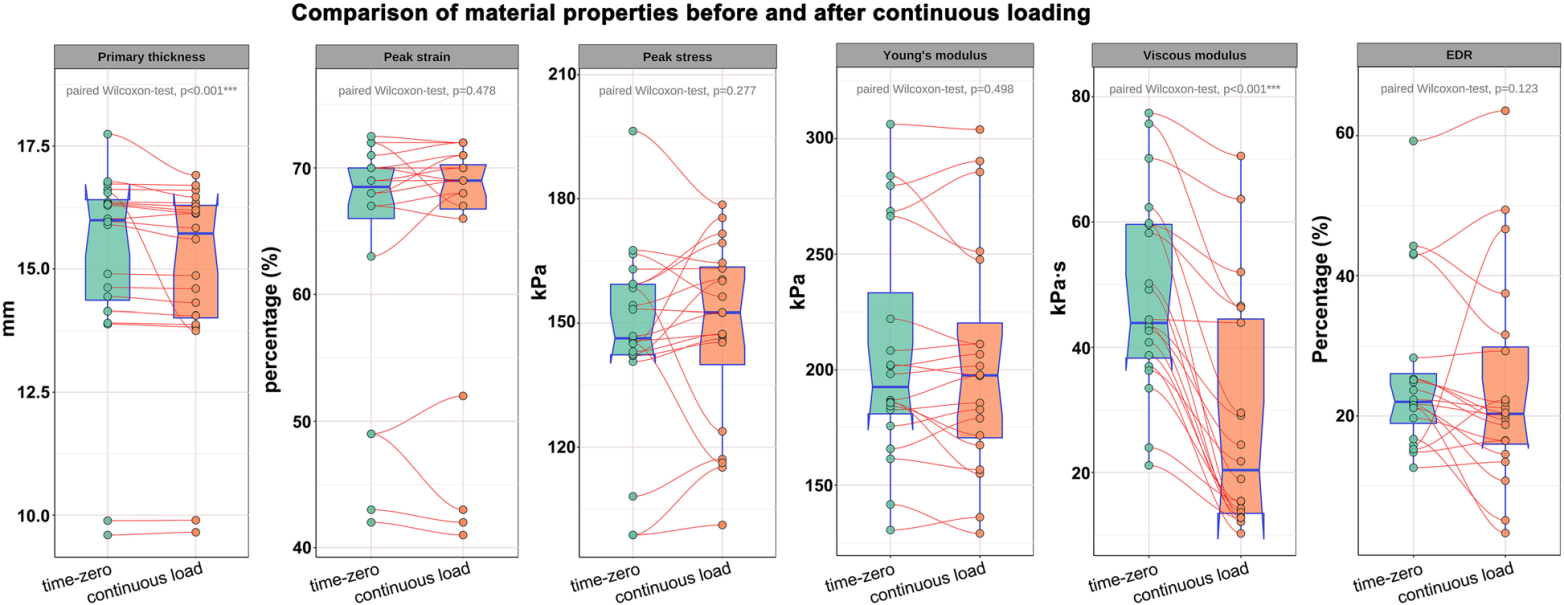
Standardized paired box plot comparing the structural and mechanical properties before and after continuous loading. Following continuous compressive loading, the viscous constant was significantly deceased (*p* < 0.01**)

Using the DFIS incorporated with concurrent force plate, time-dependent strain and stress applied to heel pad were synchronously obtained. A representative stress-strain curve depicting a cycle of loading and unloading is shown in Fig. 2. The summaries of the structural and biomechanical properties of plantar soft tissue at heel were presented in Table 1. The primary thicknesses were different at time zero (median:15.99 mm; range:9.60 ~ 17.74 mm) and following continuous loading (median:15.72 mm; range:9.65 ~ 16.91 mm) (*p* < 0.001). The peak strain (median: 0.685 vs. 0.69; *p* = 0.478) and peak stress (median: 146.34 vs. 152.515 kPa; *p* = 0.277) were statistically similar before and after continuous loading. After continuous loading on heel pad, the elastic modulus was demonstrated to be consistent with that at time zero (median: 192.55 vs. 197.585 kPa; *p* = 0.498), while the viscous modulus was significantly decreased (median: 43.9 vs. 20.37 kPa•s; *p* < 0.001). EDR was not significantly changed after continuous loading (median: 22.02% vs. 20.31%; *p* = 0.123). Figure 3 shows the standardized paired box plot comparing the structural and mechanical properties before and after continuous loading.

**Table 1:** Summaries of the material properties of plantar soft tissue at heel

Properties	Time zero			Following continuous loading		
	Left side	Right side	Combined	Left side	Right side	Combined
Primary thickness (mm)	15.43 (9.60 ~ 16.73)	15.26 (9.89 ~ 16.36)	15.40 (9.60 ~ 16.73)	15.35 (9.65 ~ 16.70)	15.10 (9.90 ~ 16.35)	15.24 (9.65 ~ 16.70)
Peak strain	0.68 (0.67 ~ 0.73)	0.70 (0.69 ~ 0.73)	0.70 (0.67 ~ 0.73)	0.69 (0.68 ~ 0.72)	0.70 (0.65 ~ 0.72)	0.70 (0.65 ~ 0.72)
Peak stress (kPa)	141.41 (98.74 ~ 146.88)	156.30 (145.10 ~ 196.40)	145.47 (98.74 ~ 196.73)	145.80 (101.20 ~ 160.10)	156.50 (116.20 ~ 178.60)	147.30 (101.20 ~ 178.60)
Young’s modulus (kPa)	170.70 (130.50 ~ 202.20)	199.90 (158.60 ~ 266.40)	185.20 (130.5 ~ 266.40)	180.90 (129.1 ~ 197.80)	204.20 (135.70 ~ 251.20)	184.20 (129.10 ~ 251.20)
Viscous modulus (kPa۰s)	41.00 (21.13 ~ 59.79)	42.62 (11.39 ~ 75.69)	42.03 (11.39 ~ 75.69)	13.20 (10.25 ~ 29.10)	22.31 (12.89 ~ 46.56)	15.39 (10.25 ~ 46.56)
EDR (%)	23.03 (15.82 ~ 44.25)	25.01 (12.62 ~ 59.24)	24.28 (12.62 ~ 59.24)	20.35 (10.74 ~ 46.65)	21.20 (13.45 ~ 63.57)	20.83 (10.74 ~ 63.57)

The data were presented with the median values as well as the minimum-to-maximum ranges

**Fig. 4:**
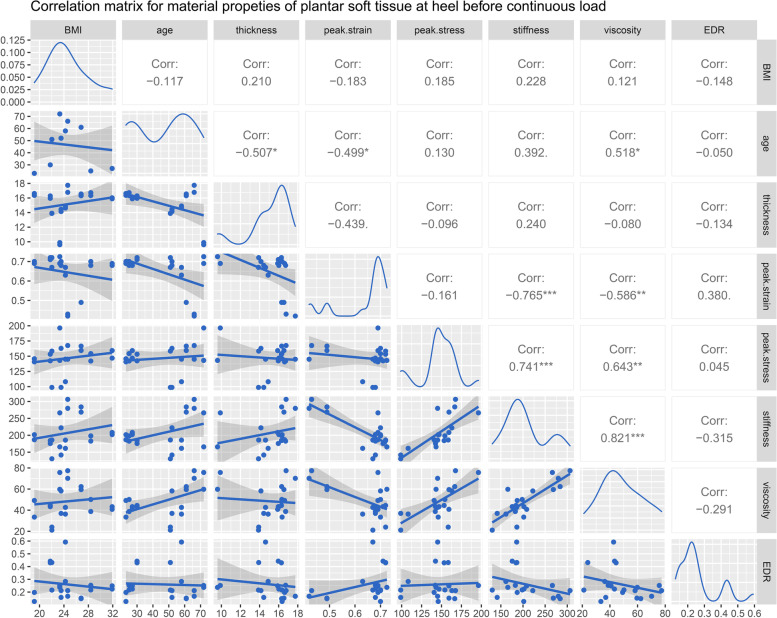
The correlation matrix for BMI, age, and the properties of heel at time zero. The values displayed in the right-upper triangle represent the Pearson’s correlation coefficients (R values). The lower-left triangle displayes the scatter plots and regression lines. The plots on the diagonal line present the distribution density of the variables in the matrix. *P* values: .*p* < 0.100, *p < 0.050, **p < 0.010, ***p < 0.001. BMI: body mass index

**Fig. 5:**
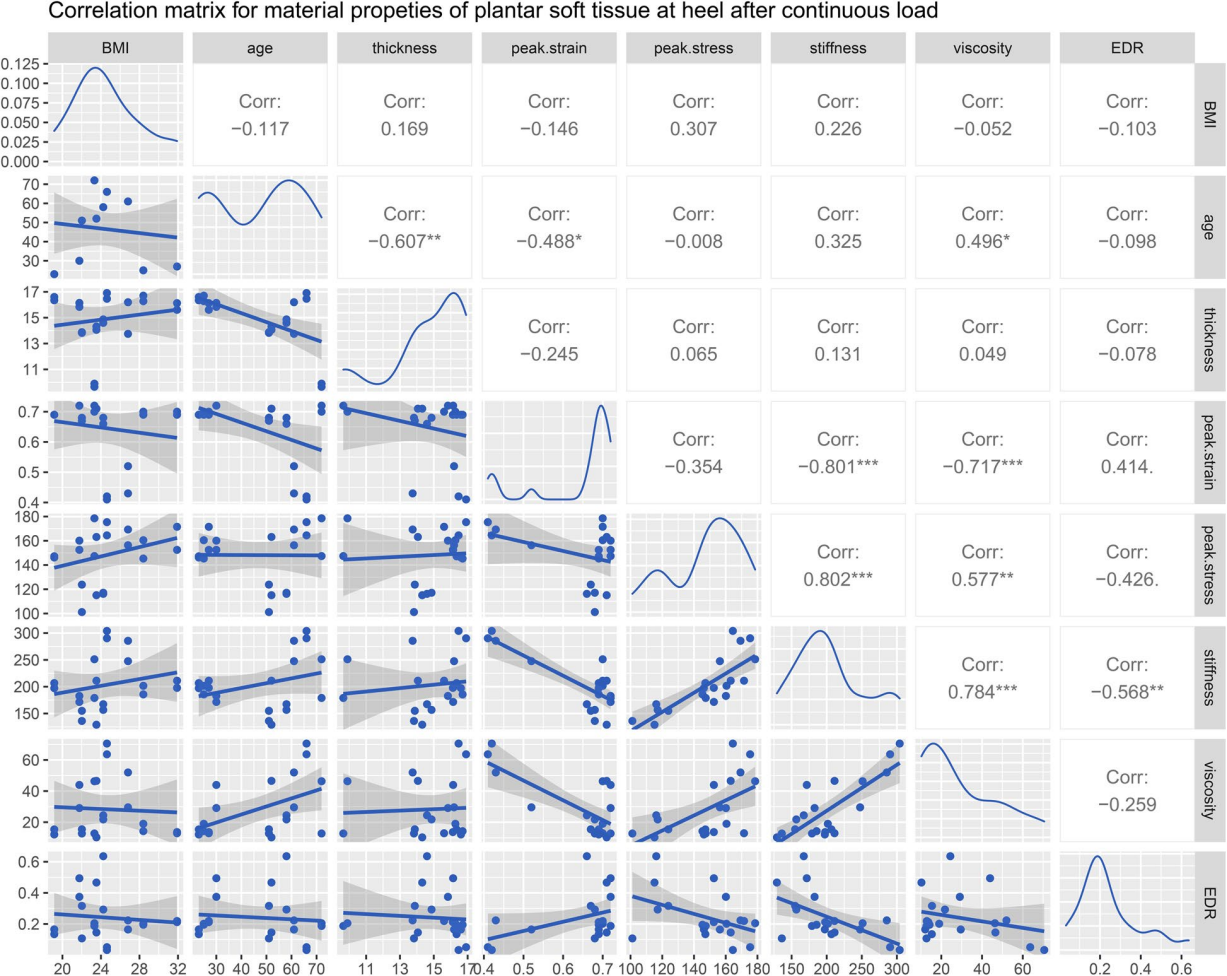
The correlation matrix for BMI, age, and the properties of heel following continuous loading. The values displayed in the right-upper triangle represent the Pearson’s correlation coefficients (R values). The lower-left triangle displayes the scatter plots and regression lines. The plots on the diagonal line present the distribution density of the variables in the matrix. P values: .*p* < 0.100, *p < 0.050, **p < 0.010, ***p < 0.001. BMI: body mass index

The correlation matrices for BMI, age, and the properties of heel at time zero and following continuous loading are available in Figs. 4 and 5, respectively. Age was demonstrated to be gently correlated with the primary thicknesses both at time zero (*R =* -0.507, Fig. 4) and following continuous load (*R =* -0.607, Fig. 5). The peak stress was significantly correlated with the elastic modulus both at time zero (*R =* 0.741, Fig. 4) and following continuous load (*R =* 0.802, Fig. 5). The peak strain was correlated with the elastic modulus at time zero (*R =* -0.765, Fig. 4) and following continuous load (*R =* -0.801, Fig. 5). The correlations between the viscous modulus and peak strain/ peak stress are similar to the above but slightly less so. It is obvious that the viscous modulus is positively correlated with the elastic modulus before (*R =* 0.821, Fig. 4) and after continuous load (*R =* 0.784, Fig. 5). Additionally, the viscous modulus is moderately correlated with age at time zero (*R =* 0.518, Fig. 4) and following continuous load (*R =* 0.496, Fig. 5). After continuous loading, moderate correlations between EDR and the elastic modulus (*R =* -0.568, Fig. 5) are presented.

## Discussion

In the current study, using the DFIS, we can capture two orthogonally frames at the same time to obtain more detailed spatial information about the tridimensional heel pad. By incorporating the DFIS system and compression force plate, the in vivo viscoelastic properties of the heel pad in healthy adults were measured in the actual gait. We found that age was negatively correlated with the primary thickness of heel pad and peak strain, and was positively correlated with viscous modulus. Additionally, repetitive loading could decrease the primary thickness of heel pad and viscous modulus.

Numerous in-vivo measurement tools, such as spherical indentation system [10, 16], instrumented pendulum [15, 28], ultrasound indentation system [17, 18, 29, 30], tissue ultrasound palpation system [31], and optical coherence tomography-based air-jet indentation system [31], have been developed in the past. These quasi-static methods, however, could not replicate the mechanical condition experienced by the heel during dynamic gait cycle. De Clercq et al. [21] firstly described a novel method using cine-radiography to evaluate the in vivo compressive strain of heel pad during running in 1994. Then, this method was expanded by Gefen et al. [22] in 2001 to include simultaneous measurements of strain rate using digital radiographic fluoroscopy and contact pressure using embedded foot-ground contact pressure display, which allows for characterization of the heel pad during the full cycle of loading and unloading in normal gait. However, as the authors specially pointed out, the one-dimension lateral X-ray projection used for measuring the heel pad thickness inevitably put some limitations on the interpretation of the results, as the true nature of the heel pad deformation is three dimensional.

The average primary/ unloaded heel pad thicknesses reported in previous studies were ranged from 11.5 to 19.1 mm in healthy adults [9, 22, 32, 33]. In addition, the primary heel pad thickness was proven to be related with the gender, age and physiques of subjects [34, 35]. Maemichia et al. [34] observed the changes on heel pad thickness associated with age, and physique in 1126 healthy Japanese, demonstrating that the thickness tends to increase from ages 1-5 (male: 10.5 ± 1.6 mm; female: 9.6 ± 1.4 mm) to 30-44 years (male: 15.8 ± 2.7 mm; female: 13.9 ± 2.1 mm) and decrease from ages 30-44 to 80-96 (male: 14.2 ± 2.7 mm; female: 11.9 ± 4.0 mm) years, and the males had higher thickness than females in the corresponding age groups. What’s more, the thickness of heel pad in males is associated with the body mass and height. In our results, the median primary thicknesses at time zero and following continuous compressive loading were 15.99 and 15.72 mm respectively. It could be speculated that the inconsistency among studies may be due to the biases caused by differences on race, age, BMI, and so on, as small sample size was reported in these researches. Similar with the results in Maemichia et al. [34], we demonstrated a negative correlation between age and primary thickness both at time zero (*R =* -0.507) and after continuous load (*R =* -0.607).

The elastic modulus measured in the current study (192.55 and 197.585 kPa at time zero and following continuous loading) was similar with the result in Gefen et al .[22] (175 kPa), which was also derived from in vivo measurement as we did. However, Ledouxa et al .[5] reported a elastic modulus as high as 830 ± 30 kPa for the heel pad, basing on in-vitro compressive test, which is more than four folders of our results. The significant difference between in-vitro and in-vivo mechanical testing has been proven in previous studies, demonstrating a six-time higher stiffness and a three-time lower energy dissipation rate for in vitro testing [13–15]. Thus, with the aim of reinstating an actual mechanical condition during gait, the current study presented a more practicable approach to involve the whole body in measurement.

It is of particular importance to evaluate the time-dependent behaviour (i.e., viscous properties) of heel fat pad, as it has been widely recognized as the major origin of the ability of shock absorption at heel strike [36]. What’s more, the modifications on viscous properties may be even more sensitive to pathological conditions, such as diabetes, than other commonly evaluated mechanical properties (such as elasticity) [37]. Using our novel system, in consequence, clinicians could easily obtain the viscous parameter to assist the diagnoses and interventions of pathological states at heel. In the current study, we calculated two significantly different viscous modulus at rest (median: 43.9, range: 21.13 ~ 75.69 kPa۰s) and fatigue (median: 20.37, range: 10.25 ~ 70.535 kPa۰s) statuses, compared to a viscosity constant of 22 kPa۰s in Gefen et al .[22]. The viscous modulus at fatigue status in our results was closed to that of the Gefen et al. [22]. It could be speculated that the measurement condition was similar with the fatigue status following 15 min of sustaining standing or wandering that performed in the current study, as the authors of the previous study have taken their subjects to train on the platform prior to data acquisition. In our study, to test time-zero mechanical properties, the subjects were required to keep their foots on relax condition and free of loading for one hour. Thus, the viscous modulus at rest was much higher than that at fatigue status, demonstrating that the loading history of heel fat pad could obviously impact the viscous property.

This study, nevertheless, has some limitations. As a pilot study, the small sample size, inevitably, would bring in potential risk of selection bias. Then, the strain rate applied to the heel pad has been widely proven to obviously impact the mechanical properties of heel pad [5, 10, 12]. While in the stance phase of gait, it is non-possible to precisely control the strain rate as that performed in in-vitro machine-based loading. To overcome this problem, subjects were trained prior to measurement to ensure an approximate gait velocity of 1.0 m/s.

## Conclusions

A novel technique using DFIS incorporated with simultaneous contact force plate for measuring the in vivo viscoelastic properties of the heel pad in the actual gait was developed. Utilizing the present approach, clinicians could accurately evaluate the in-vivo viscoelastic characteristics of heel fat pad during natural gait cycle. Age was negatively correlated with the primary thickness of heel pad and peak strain, and was positively correlated with viscous modulus. Additionally, repetitive loading could decrease the primary thickness of heel pad and viscous modulus.

## Data Availability

The datasets used and/or analyzed during the current study are available from the corresponding author on reasonable request.

## Abbreviations

DFIS: dual fluoroscopic imaging system
EDR: energy dissipation rate
BMI: body mass index
